# Hepatitis C Virus Vaccine Research: Time to Put Up or Shut Up

**DOI:** 10.3390/v13081596

**Published:** 2021-08-12

**Authors:** Alex S. Hartlage, Amit Kapoor

**Affiliations:** 1 Center for Vaccines and Immunity, Abigail Wexner Research Institute at Nationwide Children’s Hospital, Columbus, OH 43205, USA; Alex.Hartlage@osumc.edu; 2 Medical Scientist Training Program, College of Medicine and Public Health, The Ohio State University, Columbus, OH 43205, USA; 3 Department of Pediatrics, College of Medicine and Public Health, The Ohio State University, Columbus, OH 43205, USA

**Keywords:** hepatitis C virus, *Hepacivirus*, vaccine, animal model, controlled human infection model

## Abstract

Unless urgently needed to prevent a pandemic, the development of a viral vaccine should follow a rigorous scientific approach. Each vaccine candidate should be designed considering the in-depth knowledge of protective immunity, followed by preclinical studies to assess immunogenicity and safety, and lastly, the evaluation of selected vaccines in human clinical trials. The recently concluded first phase II clinical trial of a human hepatitis C virus (HCV) vaccine followed this approach. Still, despite promising preclinical results, it failed to protect against chronic infection, raising grave concerns about our understanding of protective immunity. This setback, combined with the lack of HCV animal models and availability of new highly effective antivirals, has fueled ongoing discussions of using a controlled human infection model (CHIM) to test new HCV vaccine candidates. Before taking on such an approach, however, we must carefully weigh all the ethical and health consequences of human infection in the absence of a complete understanding of HCV immunity and pathogenesis. We know that there are significant gaps in our knowledge of adaptive immunity necessary to prevent chronic HCV infection. This review discusses our current understanding of HCV immunity and the critical gaps that should be filled before embarking upon new HCV vaccine trials. We discuss the importance of T cells, neutralizing antibodies, and HCV genetic diversity. We address if and how the animal HCV-like viruses can be used for conceptualizing effective HCV vaccines and what we have learned so far from these HCV surrogates. Finally, we propose a logical but narrow path forward for HCV vaccine development.

## 1. Introduction

Chronic infections caused by the hepatitis C virus (HCV), a bloodborne RNA virus that belongs to the genus *Hepacivirus* of the *Flaviviridae* family, are a leading cause of severe progressive liver diseases and a significant public health threat. Transmission occurs mainly via infected blood exposure in setting of injection drug use, contaminated medical products, or accidental needle stick, although sexual and vertical transmission can occur as well. An estimated 71 million people are infected worldwide, and at least 400,000 die annually from HCV-related liver complications, including cirrhosis, end-stage liver disease, and hepatocellular carcinoma [1,2]. Within the United States, HCV is a leading cause of mortality by an infectious agent even exceeding that of HIV and tuberculosis combined, with transmission rates rising due to a resurgence in injection drug use fueled by an opioid epidemic [3,4].

Since its initial discovery by Choo and colleagues in 1989 as the principal cause of transfusion-associated non-A, non-B hepatitis [5], remarkable advancements have been made in our understanding, prevention, and treatment of HCV-associated disease. This includes the development of rapid molecular testing to identify infected persons, improvements in blood screening protocols, generation of efficient in vitro culture systems, and, most recently, the development of highly effective direct-acting antiviral (DAA) agents that cure >95% of treated infections. Indeed, the story of HCV research has been one of incredible success, evidenced by the recent awarding of the Nobel Prize in Medicine to Michael Houghton, Charles Rice, and Harvey Alter for their role in HCV discovery and characterization [6]. 

Despite these many successes, HCV research has unfortunately yet to deliver on one of its principle promises critical to achieving global eradication: the development of a preventive vaccine. This failure is not due to a lack of trying, as significant research has been devoted to understanding the adaptive immune responses associated with spontaneous resolution of infection in the minority of exposed individuals, with the hope of translating this knowledge into the design of effective vaccines. These efforts have yielded fundamental insights into the nature of protective HCV immunity, particularly the importance of T cells to achieving viral clearance. However, despite this knowledge, only a single vaccine candidate has successfully progressed to phase II clinical efficacy testing in humans. This recently concluded trial, which used recombinant viral vectors to stimulate pan-genotypic T cell responses against HCV nonstructural proteins, notably failed to prevent chronic infection in a cohort of high-risk injection drug users despite substantial preclinical evidence supporting its potential effectiveness. The failure of this highly anticipated trial raises serious concerns about our understanding of HCV immunity and the future of HCV vaccine research moving forward.

This review discusses our current understanding of HCV adaptive immunity, the knowledge gaps and barriers to vaccine development, and possible strategies for testing new vaccine concepts. In particular, we discuss the recent development of new HCV-like virus surrogates as well as the potential use of a controlled human infection model (CHIM) for the evaluation of future vaccine candidates. 

## 2. Need for and Feasibility of an HCV Vaccine 

Because of the remarkable efficacy of DAA treatment, the WHO has now set a target goal to eliminate HCV by year 2030 [1]. Since HCV disease often takes years to decades to develop [2] and therapeutic termination of infection significantly reduces the risk of liver-related complications [7,8], intervening with these agents represents a conceptually plausible strategy to reduce, and possibly even eradicate, HCV-related disease [9]. However, significant barriers to HCV treatment exist that are likely to hinder global elimination efforts in absence of a preventive vaccine. These include high cost of therapy, inadequate infection surveillance programs, and poor treatment adherence within difficult-to-treat patient groups [10,11]. Additionally, individuals cured by DAA therapy remain immunologically susceptible to HCV reinfection [12,13], which could complicate elimination of the virus from high-risk populations, such as injection drug users. Thus, there is a strong likelihood that a preventive vaccine, effective in HCV-naïve and/or DAA-cured individuals, will ultimately be needed to help achieve global elimination goals [9,10,11]. 

Though progress in developing an effective HCV vaccine has been regrettably slow, several lines of evidence highlight its potential feasibility. First, during the acute phase of HCV infection, initial control of viremia coincides with the emergence of virus-specific B and T cell responses in blood [14,15,16]. Long-term maintenance of these responses, particularly CD4 T cell help, is strongly correlated with spontaneous resolution of infection [17,18,19,20], which occurs in approximately 30% of HCV-naïve individuals. Second, natural clearance of HCV infection generates immunity that affords improved virological control upon reinfection and significantly reduces the risk of HCV chronicity [21,22,23,24], including occasionally against heterologous strains [25,26]. Indeed, prospective studies of injection drug users who spontaneously resolved prior infection demonstrated a 60% reduction in the rate of HCV persistence following virus re-exposure [27,28]. Thus, a vaccine that generates immunity equivalent to that conferred following natural HCV infection would be expected to be reasonably efficacious. However, because naturally acquired HCV immunity is not always reliable in preventing chronic reinfection, most notably against heterologous strains [23], vaccine platforms that stimulate novel forms of immunity should be pursued.. 

## 3. Importance of T Cells to HCV Control

A substantial body of evidence implicates a dominant role for T cells in mediating protection against HCV. Primary infection is characterized by a prolonged period of viral replication lasting 8–12 weeks before sharply declining in association with development of blood HCV-specific CD4 and CD8 T cell responses [29]. HCV viremia may oscillate at low levels for a period of weeks to months before it is ultimately terminated or rebounds to persistently high levels. Importantly, failure to eliminate HCV during this critical period is associated with a functional loss of HCV-specific T cell immunity [15]. In particular, failure to maintain HCV-specific CD4 T cell help is a principal feature of infections that eventually persist [17,18,19,20]. The importance of T cells to HCV control is further supported by a number of genetic association studies linking acute clearance with expression of MHC class I and II alleles [30,31,32,33]. 

Following the resolution of primary HCV infection, memory CD4 and CD8 T cells are generated that survive for years after apparent virus clearance. Indeed, rapid control of secondary HCV infections, observed in humans and chimpanzees, is temporally associated with the accelerated recall of these populations [21,24,34]. In immune chimpanzees, transient antibody-mediated depletion of memory CD4 or CD8 T cells provided direct evidence that these subsets are required for durable protection. Removal of CD8 T cells resulted in a prolonged period of viral replication, which was eventually terminated following recovery of HCV-specific CD8 T cells in the liver [24]. Conversely, depletion of CD4 T cells resulted in low levels of persistent viremia lasting several months [34]. Interestingly, failure to eliminate the virus in this setting was associated with selection of viral escape mutations in dominant MHC class I epitopes that impaired recognition by HCV-specific CD8 T cells in vitro, suggesting that CD8 T cells are the major controllers of HCV infection after virus re-exposure and that effective antiviral activity from this subset is critically dependent upon sufficient CD4 T cell help. Given these apparent vital roles, T cells remain a principal target of HCV vaccination strategies [35]. 

## 4. A Possible Role for Antibodies 

Unlike T cells, there is still significant uncertainty about the protective capacity of antibodies during HCV infection. This uncertainty stems partly from the paradoxical observation that patients with underlying B cell defects can resolve HCV infection in the absence of presumable antibody activity [36]. Likewise, chimpanzees often lack HCV neutralizing antibody responses against the E1 and E2 envelope glycoproteins despite a relatively high clearance rate in comparison to humans [37]. 

Despite these observations, there is accumulating evidence, particularly recent, that neutralizing antibodies may in fact be protective. In humans, for example, spontaneous clearance of acute HCV infection has been demonstrated to coincide with the development of serum broadly neutralizing antibodies (bnAbs) that recognize multiple heterologous strains [38,39,40]. Interestingly, in a single patient with chronic HCV infection, delayed clearance was correlated with the late development of bnAbs [41]. During secondary infections of immune individuals, accelerated control of viremia has similarly been linked to the rapid recall of serum bnAb responses [28]. 

Studies in animal models of HCV infection also suggest a role for antibody-mediated protection. Passive transfer of polyclonal immunoglobulins from the serum of a chronically infected donor prevented infection in human liver chimeric mice by both autologous [42] and heterologous [43] HCV. Similar results were obtained in chimpanzees, where the transfer of human immunoglobulin prevented infection by the homologous but not heterologous virus [44]. Notably, treatment of chronically infected chimpanzees with immune serum or a broadly reactive anti-E2 monoclonal antibody reduced HCV viremia [45,46], whereas infusion of bnAbs led to viral resolution in human liver chimeric mice [47]. These studies suggest that antibodies provide at least partial protection against HCV and that sterilizing immunity might be achievable in theory if bnAbs with coverage against multiple heterologous strains can be raised to sufficient levels. 

## 5. HCV Subversion of Adaptive Immune Responses

HCV’s success as a human pathogen is a direct result of its ability to disrupt host B and T cell responses to establish a lifelong state of persistent viral replication. Although the mechanisms that enable persistence are incompletely defined, detailed analyses of individuals with chronic versus resolving infection, including studies conducted in experimentally infected chimpanzees, have revealed critical insights into how HCV distorts protective immune responses to prolong the infection. 

Because virus-specific CD8 T cells circulate in blood at measurable frequencies during chronic HCV infection, mechanisms impairing this subset are the best described. To date, two primary mechanisms are recognized to interfere with HCV-specific CD8 T cell functions in vivo [14,48]: (1) mutational escape at MHC class I epitopes; and (2) immune dysfunction or exhaustion, a terminal differentiation state characterized by progressive loss of antiviral effector functions, lack of memory cell formation, and persistent upregulation of multiple co-inhibitory receptors, such as programmed cell death-1 (PD-1) [49]. The factors that sustain effective CD8 T cell immunity and prevent these processes during spontaneous clearance of acute HCV infection are unknown but likely related to the ability of CD4 T cells to maintain critical helper functions [34,50]. It is important to note that exhaustion is primarily restricted to CD8 T cells targeting intact (i.e., non-escaped) class I epitopes [51], since these populations receive persistent antigenic stimulation necessary to drive the exhaustion program [49]. Whether these exhausted CD8 T cell populations are permanently damaged or able to recover via therapeutic or vaccine intervention after DAA cure and protect against future infection is uncertain and an important question with practical implications for HCV eradication [9]. 

As noted above, premature loss of the virus-specific CD4 T cell response is the earliest and strongest predictor of acute HCV infections that will become chronic. Although the mechanism(s) by which HCV triggers failure of CD4 T cells has not yet been elucidated, several key characteristics that define a successful versus unsuccessful response have been delineated. Regardless of infection outcome, the acute phase of infection is characterized by a remarkably broad HCV-specific CD4 T cell response targeting on average 10 MHC class II epitopes [19]. These cells express high levels of the co-inhibitory receptors PD-1 and cytotoxic T-lymphocyte-associated protein 4 (CTLA-4), classical markers of T cell activation and exhaustion, and show no ostensible difference in their ability to produce canonical antiviral cytokines [20]. As HCV infection progresses to chronicity, HCV-specific CD4 T cells rapidly lose their ability to proliferate and decline dramatically in frequency while maintaining expression of co-inhibitory receptors [18,19,20]. Reciprocally, termination of infection coincides with loss of PD-1 and CTLA-4 expression on HCV-specific CD4 T cells and upregulation of the memory cell marker CD127 [20]. An indisputable role for mutational escape at MHC class II epitopes has not been demonstrated [52,53,54], although evidence of CD4 T cell selection pressure has been found at the population level [55]. It is not yet clear whether the defect in HCV-specific CD4 T immunity is permanent or reversible, although spontaneous recovery of responses in chronically infected mothers shortly following childbirth signifies a capacity for restoration [56]. Notably, a recent study showed that HCV-specific CD4 T cells with a B cell-supportive T follicular helper cell signature are maintained in absence of persistent antigenic stimulation following DAA cure [57]. How this population responds to antigen re-exposure has not yet been defined but may require targeted restoration to overcome HCV silencing mechanisms and prevent persistent infection upon virus re-exposure. 

Although the overall importance of B cells to spontaneous HCV clearance is unresolved, the virus employs multiple strategies to evade recognition by the humoral response. In particular, HCV circulates in complex host-derived lipoproteins which, combined with heavy glycosylation of the envelope glycoproteins, helps shield it from the action of host neutralizing antibodies and may contribute to their delayed induction [58,59]. Furthermore, most antibodies commonly target highly mutable areas of the genome, known as hypervariable regions, that evolve rapidly to counteract immune pressure [60,61]. Finally, bnAbs that target more heavily conserved regions of the envelope glycoproteins can be blocked by non-neutralizing antibodies that circulate at higher frequency [62]. These mechanisms could be overcome, in theory, by designing vaccine antigens that target critically conserved domains and induce bnAbs of sufficient potency and duration. Of course, the requirements for inducing such a response in patients cured of persistent infection, where existing humoral immunity may impede induction of new antibodies, are ill-defined and may present additional unknown hurdles. 

## 6. HCV Genetic Diversity and Implications for Vaccine Design

HCV possesses extraordinary genetic diversity and is currently classified into seven major phylogenetic lineages termed genotypes (that differ by more 30% in sequence), along with numerous recognized subtypes [63]. Each HCV genotype encodes a large polyprotein that is processed by host and viral-derived proteases into three structural (core, E1, E2) and seven (p7, NS2–NS5) nonstructural proteins. In infected individuals, HCV circulates as a viral “quasispecies” comprised of multiple closely related yet distinct viruses. Because of the inherent infidelity of HCV’s viral RNA polymerase, which generates mutations at a rate ten-fold higher than that for HIV or hepatitis B virus [64], these populations can readily evolve to escape recognition from host neutralizing antibodies and CD8 T cells. This genetic heterogeneity and capacity for rapid diversification poses a significant challenge to HCV vaccine development, where induction of a broadly reactive vaccine response capable of controlling existing and emerging variants is imperative for success. 

As mentioned above, raising effective neutralizing antibodies against HCV is a difficult undertaking since these principally target the E1 and E2 envelope glycoproteins, which are highly variable across and within known genotypes. A pan-genotypic neutralizing response could be generated in theory using antigens that mimic critically conserved domains. Still, additional improvements in structural antigen design and vaccine formulation will likely be needed before such a targeted approach. However, a recent analysis of bnAbs isolated from B cells of convalescent patients demonstrated a relatively low level of somatic hypermutations [65], so vaccine induction of such a response may be more feasible than anticipated. Regardless, there is still the uncertainty of whether such antibodies would be capable of penetrating the glycan and lipoprotein shield covering the HCV virion sufficiently to prohibit in vivo infection. 

Because HCV-specific T cells predominantly target epitopes within nonstructural proteins, which are more evolutionarily conserved across genotypes [66], vaccines designed to elicit T cell immunity should, in principle, generate broader protection than antibody-based approaches and minimize the overall risk for selecting escape variants [35]. This notion is supported by the observation that T cells induced by vaccines encoding nonstructural antigens can recognize HCV variants of shared and disparate genotypes [67,68,69]. Moreover, nonstructural proteins of several diverse HCV variants have been found to share immunodominant T cell epitopes [70], and such stretches of evolutionarily conserved amino acids can be used to design multi-genotypic immunogens [71]. Nevertheless, it is still unclear whether a purely T cell-based approach relying upon nonstructural protein-based immunogens will be sufficient to provide protective immunity in the absence of antibody support. Indeed, a meta-analysis of vaccine efficacy testing in chimpanzees revealed a protective effect of HCV structural proteins [26], which stimulate neutralizing antibodies and less so T cells. Thus, it will be of critical importance moving forward to determine whether HCV vaccines should focus on generating T cells, antibodies, or both, as this knowledge is essential for proper immunogen selection and design. 

Proper vaccine design will also depend upon HCV global distribution. Current genetic analyses of HCV diversity indicate that while genotypes 1 and 3 are highly prevalent worldwide, the remaining genotypes are primarily restricted to countries in Africa and Asia [72]. Thus, an HCV vaccine that is effective against genotypes 1 and 3 will likely have a significant effect on reducing the global burden of new HCV infections each year. Subsequently, vaccines for other less prevalent genotypes can be developed for their targeted deployment in at-risk populations. Indeed, a similar approach has proven effective in the worldwide eradication of poliovirus serotype 2 and 3 infections [73]. 

## 7. Candidate HCV Prophylactic Vaccines

A large variety of candidate HCV vaccines targeting humoral and/or cellular immunity have been evaluated for immunogenicity in preclinical animal models [35,74]. These range from traditional immunization approaches, such as whole proteins and virus-like particles, to newer DNA and recombinant viral vector-based strategies. Many of these focused on either the envelope glycoproteins for generation of neutralizing antibodies or nonstructural proteins (e.g., NS3-5B) that are strong inducers of CD4 and CD8 T cell responses, although several took dual-approaches. Very few of these candidates were evaluated for their protective efficacy in chimpanzees, with mixed results (Table 1). Of those that showed some degree of effectiveness in preventing chronic infection in the chimpanzee model, only two have progressed to human clinical trial testing. 

The first is an antibody-based vaccine originally developed by Chiron Corporation (now Novartis) consisting of recombinant E1 and E2 glycoproteins from a genotype 1a virus adjuvanted with MF59 [95]. In chimpanzees, this regimen elicited a strong antibody response that protected against homologous challenge [75,96]. Against heterologous challenge, this regimen did not provide sterilizing immunity but did delay the onset of viremia and greatly attenuated the course of infection compared to unvaccinated controls [95]. In healthy human volunteers, immunization elicited strong HCV-specific CD4 T helper responses and, in some recipients, cross-genotype neutralizing antibodies [78,79]. Despite these encouraging results, follow-up trials to evaluate efficacy have yet to proceed [97]. 

The second candidate is a T cell-based vaccine developed by Okairos Corporation (now Glaxo Smith Kline) that consists of a recombinant chimpanzee adenovirus (serotype 3) prime and MVA boost encoding the NS3-5B proteins from a genotype 1b virus. Support for this approach was first provided in chimpanzees, where immunization with an adenovirus prime followed by a plasmid DNA boost induced long-lived CD4 and CD8 T cell responses against nonstructural proteins [98]. The heterologous challenge of animals resulted in a rapid recall of memory T cell responses and significant suppression of acute-phase viremia compared to historical controls [68]. In healthy human volunteers, this approach stimulated broad, multi-potent CD4 and CD8 T cell responses that resembled those generated upon spontaneous resolution of primary infection [69]. Based on this data, a phase II clinical efficacy trial was recently conducted in a group of high-risk injection drug users. Several aspects of this trial are notable. First, it tested the novel idea that a vaccine that engages T cell immunity alone could prevent persistent infection in the absence of neutralizing antibody responses. Second, it demonstrated the feasibility of conducting rigorous vaccine research within persons who inject drugs, an uneasy feat given the substantial resources and outreach required to maintain trial follow-up and engagement. Although the regimen stimulated HCV-specific T cell responses in the majority of vaccinees, no reduction in the incidence of chronic infection was observed [88]. The reasons for the failure of this highly anticipated trial are not yet understood. 

## 8. Novel *Hepacivirus* Models for HCV Study 

The lack of immunologically tractable animal models is a major barrier to HCV vaccine development. HCV displays a strict species tropism for humans, and the only permissive animal model is the chimpanzee [99], which is no longer available due to significant ethical concerns regarding its continued use. Several HCV mouse models have been engineered to support the viral lifecycle and can be utilized for assessing in vivo antibody activity, as highlighted above. However, these platforms currently require blockade of host immune responses to function, limiting their overall application towards HCV vaccine research [99,100]. 

Given these challenges, one reasonable solution is to instead use a closely related virus, similar in biological properties, as an in vivo surrogate. The closest genetic relative of HCV is the non-primate *Hepacivirus* (NPHV), which was first identified in 2011 and circulates naturally in domestic horses. Experimental NPHV infection of horses recapitulates several important features of HCV, including capacity to cause chronic infection, delayed seroconversion, and acute liver pathology [101,102,103]. In one study, spontaneous resolution of primary NPHV infection was shown to be associated with immune protection against reinfection, similar to HCV [103]. Despite these promising overlaps, however, the horse is not a readily available or easily tractable animal model, limiting its overall application. Furthermore, horse NPHV infection is spontaneously cleared at high frequency, which could complicate assessment of vaccine concepts with prevention of chronic infection as the primary endpoint. 

In 2014 a rodent *Hepacivirus* (RHV) with striking similarities to HCV was discovered in livers of feral Norway rats in New York City [104]. Notably, passage of an isolate into immune-competent laboratory mice [105] and rats [106] resulted in robust hepatotropic viral infection. Viremia was cleared after several weeks in mice via T cell-dependent mechanisms, but spontaneously persisted at high levels in rats, the virus’ natural host. Despite efforts to further adapt RHV to mice, chronic infection could only be established in strains lacking components of innate or adaptive immunity [105], precluding the use of mice for vaccine concept testing. In contrast, further characterization of infection in rats revealed HCV-like patterns of liver inflammation, innate immune sensing, spontaneous T cell dysfunction, and sensitivity to direct-acting antiviral treatment [106,107]. 

The utility of the rat *Hepacivirus* infection model for HCV vaccine research was recently demonstrated by two studies evaluating the effectiveness of T cell immunization in preventing RHV persistence. Vaccination of rats with adenoviral vectors expressing the RHV NS3-5B proteins, akin to the human HCV vaccine candidate, generated CD4 and CD8 T cell responses and significantly reduced the incidence of chronic infection after homologous challenge [108,109]. The protective effect was T cell-dependent, as depletion of CD4 or CD8 T cells immediately prior to challenge resulted in a substantially attenuated infection course [108]. Interestingly, inclusion of the E1 and E2 glycoproteins as vaccine targets resulted in increased protection from viral persistence [109], although the exact mechanism of this effect is not yet understood. In a recent follow-up study, it was demonstrated that challenge with an RHV strain encoding mutations in dominant MHC class I epitopes that impaired RHV-specific CD8 T cell function in vitro substantially reduced vaccine protection in naïve rats [107], highlighting the importance of cross-reactivity between vaccine-induced T cell responses and infecting virus strain. Interestingly, however, vaccinated rats who had already cleared homologous infection were found to be protected against subsequent challenge with mutated virus. This observation suggests that vaccine-generated immunity can be enhanced to prevent infection by genetically diverse *Hepacivirus* strains. However, the mechanism of improvement is not yet clear and, in particular, whether humoral immunity played any contributory role. 

Together, these studies highlight the overall ease and potential utility of rat RHV infection for assessing mechanisms of HCV-like immunity and vaccine concept testing. While in vitro models for measuring anti-RHV neutralizing antibodies are not yet available, efforts are currently underway [110] and could be instrumental in helping resolve the obscure role of B cells in protection against chronic HCV infection. For example, neutralizing antibody responses could be raised via vaccination against the RHV E1 and E2 glycoproteins, with subsequent viral challenge to assess for protection. Mechanistic determination of efficacy could then be swiftly determined via sera transfer and depletion studies. Furthermore, because RHV is susceptible to direct-acting antiviral treatment [106,107,110], whether vaccination is effective after therapeutic cure, a key question with broad implications for chronic viral infections in general, could be addressed. 

There are, however, several limitations to the rat RHV model that should be noted. First, there is currently only one strain of RHV available for experimental study, precluding the assessment of true heterologous immunity following vaccination. This could be potentially addressed by further isolation of genetically diverse RHV strains from wild rats or by selecting strains that naturally acquired vaccine resistance [107]. Similarly, it is unknown whether RHV circulates as a quasispecies during chronic infection like HCV, so additional strategies to help emulate this may need to be explored. Second, access to immunological reagents is limited for rats and may hinder more detailed studies aimed at understanding mechanisms of immune dysfunction and vaccine failure. Finally, it is difficult to predict how findings in the rat RHV model will ultimately translate to humans; failure of the recent T cell vaccine trial in humans certainly supports this concern. 

## 9. Considerations for Using a Controlled Human Infection Model

The lack of robust animal models for HCV combined with the scientific and practical challenges of conducting large-scale trials within at-risk populations has fueled ongoing discussions of initiating a controlled human infection model (CHIM) to accelerate vaccine development. Because curative treatments for HCV have been reliably established through direct-acting antivirals, such an approach is now reasonably plausible. In such studies, healthy adult volunteers would be immunized with a promising vaccine candidate or placebo and then exposed to a well-characterized strain of HCV to assess efficacy. Although acute HCV infection is typically benign, study participants who develop clinically concerning findings or chronic viremia could be rapidly treated to prevent long-term morbidity. 

The potential benefits of such an approach are significant. Promising HCV vaccine candidates could carefully and expeditiously be identified for efficacy in a highly controlled scientific setting, and then rapidly advanced for further testing in high-risk populations if deemed necessary. This would minimize the tremendous cost and practical challenges associated with conducting studies in people who inject drugs, who require extensive risk-reducing strategies, and often present with significant social vulnerabilities that complicate long-term follow-up. By consequence, the bottlenecks of HCV vaccine development would be effectively sidestepped, generating a rich pool of potential candidates with strong scientific support. 

Despite these potential advantages, the prospect of a controlled human infection model for HCV raises multiple scientific and safety concerns. From a scientific perspective, it is not yet clear whether a controlled human infection would adequately simulate a natural HCV exposure event. Inoculation of study participants with a culture-derived HCV strain is an unviable approach as these possess multiple adaptive mutations that enhance in vitro replication and may have unintended effects upon human infection. Use of plasma-derived virus from infected individuals would be required, mainly since this contains the aforementioned HCV quasispecies; however, the risk of transmitting additional infectious pathogens could confound results. Furthermore, how route and dose might influence the infectious course in a controlled setting compared to natural exposure is unclear. Finally, it is unknown whether results obtained from healthy participants can be reliably translated to high-risk populations, who appear to have an intrinsically altered response to HCV vaccination [88]. 

In terms of safety, the major risks of controlled human infection stem primarily from the necessarily long duration of vaccine study. Since virtually all HCV disease occurs during chronic infection, the primary measure of HCV vaccine effectiveness is whether it prevents development of persistent viremia, which is not reliably established until 3–6 months after virus exposure. HCV vaccine studies therefore require an extensive follow-up period during which participants could unknowingly transmit the virus, for instance to sexual partners. Potential loss of study participants during this timeframe is similarly a major concern and one that would have to be properly safeguarded against to ensure swift treatment in case chronic infection were to occur. While the potential for resistance to direct-acting antiviral treatment exists and must be acknowledged, the overall risk is minimal and can be effectively avoided by infecting with a strain that is known to be sensitive.

## 10. A Narrow Path forward for HCV Vaccine Development

**Figure 1:**
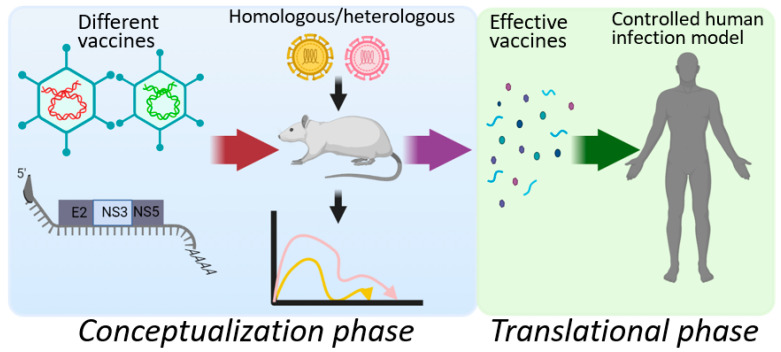
A rational approach to HCV vaccine development and testing.

The limited progress achieved for HCV vaccine development raises serious doubts about the future of the field. While widescale availability of therapeutic cure can help mitigate ongoing transmission and viral prevalence, development of an effective vaccine will likely be required to achieve global elimination and therefore should continue to be pursued. However, the exact strategy that should be undertaken to broaden the pool of HCV vaccine candidates and the speed at which they are evaluated in humans remains to be resolved. In the absence of a true HCV animal model, the two most promising options for testing new HCV vaccine concepts, as highlighted above, are via animal *Hepacivirus* models that mimic human HCV infection and through controlled human infection studies. The former offers a rapid pathway for evaluating novel delivery vectors and vaccine targets with potential for mechanistic dissection, while the latter offers an accelerated and far simpler avenue for assessing safety and efficacy prior to engaging in the more complicated route of high-risk population testing. Taking advantage of both options, a rational approach to HCV vaccine design can be envisioned, composed of distinct conceptualization and translational phases of development (Figure 1). During conceptualization, varied vaccine strategies could be engineered and evaluated in the rat *Hepacivirus* model for their ability to prevent diverse *Hepacivirus* challenges. Successful candidates could then be advanced for translational testing in a relatively small number of volunteers in a controlled human infection model. Although this is a very narrow channel for moving HCV vaccine development forward, it has the potential to generate the quality and quantity of candidates needed to make meaningful progress in this arena and should be aggressively pursued. 

## 11. Conclusions

The development of an effective HCV vaccine remains a principal objective in the effort to control and eventually eradicate global infections. Inability to deliver on this goal despite decades of in-depth research highlights a pressing need to adopt new investigational strategies that can accelerate progress in this arena. The emergence of new animal *Hepacivirus* models that recapitulate HCV infection represent an intriguing avenue for identifying mechanisms of immune control and testing novel vaccine concepts. Furthermore, the availability of curative antiviral treatments has now made the prospect of a controlled human infection model with HCV a realistic possibility, although continued deliberation regarding safety and practicality is required before fully embracing such an approach. Regardless, bold strategies are needed if the goal ofa vaccine is to be realized or forever remain a blotch on an otherwise impressive record of HCV research and discovery.

## Figures and Tables

**Table 1:** Candidate HCV prophylactic vaccines.

Vaccine Type	Antigen	Delivery/Adjuvant	Species	Immunogenicity	Challenge	Clinical Trial	Outcome	Notes	Ref.
Recombinant protein	GT1a E1E2	MF59 or MF75	Chimpanzees (*n* = 21)	Anti-E1E2 antibodies	Homologous	-	5 aviremic, 14 resolved, 2 chronic	Protected against acute and chronic infection.	[75,76]
	GT1b E1	Alum	Humans (*n* = 20)	Anti-E1 antibodies, CD4 T cells	-	Phase I	-	-	[77]
	GT1a E1E2	MF59	Humans (*n* = 60)	Neutralizing antibodies, CD4 T cells	-	Phase I	-	-	[78,79]
	GT1a core	ISCOMATRIX	Humans (*n* = 30)	CD4 and CD8 T cells	-	Phase I	-	-	[80]
	GT1b E1 or E2△HVR-1	Alum	Chimpanzees (*n* = 4)	CD4 T cells; neutralizing antibodies in E1 recipients	Heterologous GT1b	-	2 resolved, 2 chronic	E1 but not E2 vaccine recipients protected against chronic infection.	[81]
	GT1a E1E2	RIBIs	Chimpanzees (*n* = 1)	Anti-E1E2 antibodies, T cells	Homologous	-	1 chronic	Delayed infection by 3 weeks but did not prevent persistence	[82]
Recombinant protein and peptide	GT2 E1E2 and HVR-1 peptides	Complete and incomplete Freund’s	Chimpanzees (*n* = 1)	Anti-E1E2 and HVR-1 antibodies	Homologous		1 resolved	Protected against chronic infection.	[83]
DNA	GT1a E2	None	Chimpanzees (*n* = 2)	Anti-E2 antibodies, T cells	Homologous	-	2 resolved	Protected against chronic infection.	[84]
Viral vector	GT1b NS3-NS5B	Ad6/ChAd3	Humans (*n* = 41)	Heterotypic CD4 and CD8 T cells	-	Phase I	-	-	[85]
	GT1b NS3-NS5B	Chad3/MVA	Humans (*n* = 55)	Heterotypic CD4 and CD8 T cells	-	Phase I	-	-	[69]
	GT1b NS3-NS5B fused to MHC class II-associated invariant chain	Chad3/MVA	Humans (*n* = 17)	Heterotypic CD4 and CD8 T cells		Phase I	-	-	[86]
	GT1b core, E1, E2, p7, NS2, NS3	Vaccinia	Chimpanzees (*n* = 4)	T cells	Homologous	-	4 resolved	Protected against chronic infection.	[87]
	Gt1b NS3-NS5B	Chad3/MVA	Humans (*n* = 548)	T cells	-	Phase I/II	-	Reduced peak viremia but did not protect against chronic infection upon natural exposure	[88]
DNA and viral vector	GT1b Core-E1E2, NS3	DNA/MVA	Chimpanzees (*n* = 4)	CD4 and CD8 T cells	Homologous	-	1 resolved, 3 chronic	Reduced peak viremia but did not protect against chronic infection.	[89]
	GT1b NS3-NS5B	Ad6/Ad24 + electroporated DNA	Chimpanzees (*n* = 5)	CD4 and CD8 T cells	Heterologous GT1a	-	4 resolved, 1 chronic	Protected against chronic infection.	[68]
	GT1a NS3, NS5A, NS5B	DNA/vaccinia-expressing B7.1, ICAM-1, LFA-3; CpGs	Chimpanzees (*n* = 1)	CD4 and CD8 T cells	Homologous	-	1 chronic	Early suppression of viremia but late resurgence and eventual persistence	[90]
	GT1b Core-E1E2, NS3-NS5B	DNA/Ad5; hIL-12 expressing plasmid	Chimpanzees (*n* = 6)	Neutralizing antibodies, T cells	Homologous		1 aviremic, 1 resolved, 4 chronic	Sterilizing immunity associated with high anti-E2 neutralizing antibody response	[91]
	GT1a NS3-NS5B	DNA/Ad5; hIL-12 expressing plasmid	Chimpanzees (*n* = 2)	T cells	Homologous	-	1 resolved, 1 chronic	Early suppression of viremia	[92]
DNA and recombinant protein	GT1a Core, NS3; GT1b Core, E1E2, NS3	Alum	Chimpanzees (*n* = 2)	Anti-E1E2 antibodies, T cells	Homologous	-	1 resolved, 1 chronic	Reduced peak viremia.	[93]
Virus-like particles	GT1b Core-E1E2	AS01B (*n* = 2)	Chimpanzees (*n* = 4)	CD4 and CD8 T cells	Homologous	-	4 resolved	Protected against chronic infection.	[94]

## Data Availability

No new data were created or analyzed in this study. Data sharing is not applicable to this article.
